# Recent Advances in Mechanisms of Plant Defense to *Sclerotinia sclerotiorum*

**DOI:** 10.3389/fpls.2019.01314

**Published:** 2019-10-18

**Authors:** Zheng Wang, Lu-Yue Ma, Jun Cao, Yu-Long Li, Li-Na Ding, Ke-Ming Zhu, Yan-Hua Yang, Xiao-Li Tan

**Affiliations:** Institute of Life Sciences, Jiangsu University, Zhenjiang, China

**Keywords:** *Sclerotinia sclerotiorum*, plant defense, signaling molecules, quantitative resistance, genome-wide association study, candidate resistance genes

## Abstract

*Sclerotinia sclerotiorum* (Lib.) de Bary is an unusual pathogen which has the broad host range, diverse infection modes, and potential double feeding lifestyles of both biotroph and necrotroph. It is capable of infecting over 400 plant species found worldwide and more than 60 names have agriculturally been used to refer to diseases caused by this pathogen. Plant defense to *S. sclerotiorum* is a complex biological process and exhibits a typical quantitative disease resistance (QDR) response. Recent studies using *Arabidopsis thaliana* and crop plants have obtained new advances in mechanisms used by plants to cope with *S. sclerotiorum* infection. In this review, we focused on our current understanding on plant defense mechanisms against this pathogen, and set up a model for the defense process including three stages: recognition of this pathogen, signal transduction and defense response. We also have a particular interest in defense signaling mediated by diverse signaling molecules. We highlight the current challenges and unanswered questions in both the defense process and defense signaling. Essentially, we discussed candidate resistance genes newly mapped by using high-throughput experiments in important crops, and classified these potential gene targets into different stages of the defense process, which will broaden our understanding of the genetic architecture underlying quantitative resistance to *S. sclerotiorum*. We proposed that more powerful mapping population(s) will be required for accurate and reliable QDR gene identification.

## Introduction

*Sclerotinia sclerotiorum* (Lib.) de Bary is a devastating fungal plant pathogen with a broad host range including at least 408 described species of plant from 278 genera in 75 families (Boland and Hall, 1994). It can infect many economically important dicotyledonous crops such as oilseed rape (*Brassica napus*), edible dry bean (*Phaseolus vulgaris*), soybean (*Glycine max*), dry pea (*Pisium sativum*), chickpea (*Cicer arietinum*), peanut (*Arachis hypogaea*), sunflower (*Helianthus annuus*), lentils (*Lens culinaris*), and various vegetables, and some monocotyledonous species such as tulip (*Tulipa gesneriana*) and onion (*Allium cepa*) (Boland and Hall, 1994; Bolton et al., 2006). More than 60 names have been used to refer to the resulting diseases in agriculture (Purdy, 1979) including cottony rot, watery soft rot, drop, crown rot, blossom blight, and perhaps most common, *Sclerotinia* stem rot (SSR) or white mold. Diseases caused by *S. sclerotiorum* have a worldwide distribution and cause serious crop losses around the world. For example, in China, oilseed rape yield losses caused by SSR usually range from 10 to 20% and may be up to 80% for severe SSR outbreaks seasons (Yang, 1959; Li et al., 2006; Mei et al., 2011). In United States, annual losses caused by this pathogen have exceeded $200 million (Bolton et al., 2006). Diseases caused by *S. sclerotiorum* have traditionally been difficult to control (Bolton et al., 2006) due to lacking high level resistance in major crops, which makes it difficult to improve resistance using classical breeding methods. Disease management depends heavily on the application of fungicides, but this may cause environmental contamination, increase farming costs and may be ineffective because of the difficulties associated with the application of sprays to thick canopies and the lack of suitable forecasting methods to enable the timely application of fungicides. Currently, molecular breeding is pursued as an important strategy to control diseases caused by this pathogen. Thus, it is important for breeders to understand the molecular basis of host genetic resistance against *S. sclerotiorum*.

The molecular basis of plant — *S. sclerotiorum* interaction is complicated. *S. sclerotiorum* is an ascomycete with a reported necrotrophic lifestyle, secreting cell wall degrading enzymes and toxins, such as oxalic acid, Ss-Rhs1 and so on (Yu et al., 2017), to kill host cells and derive energy. Recent research revealed that there is a potential short biotrophic phase in the lifestyle of *S. sclerotiorum*, and a new model depicting the lifestyle transition of the pathogen from biotrophic to necrotrophic growth was proposed (Kabbage et al., 2015), which suggests a multifaceted pathogenic strategies for this pathogen. In return, plants use a range of multifaceted defense mechanisms to accurately detect and appropriately respond to the infection of this pathogen. Genetic resistance to *S. sclerotiorum* shows quantitative inheritance (Bert et al., 2002; Liu et al., 2005; Zhao et al., 2006; Perchepied et al., 2010; Fusari et al., 2012). Early studies looking for resistance loci in *B. napus* were conducted through quantitative trait loci (QTL) mapping (Zhao and Meng, 2003; Zhao et al., 2006; Yin et al., 2010; Behla, 2017; Wu et al., 2013; Wei et al., 2014). Alternatives to QTL mapping based on a biparental population with fewer recombination events, have emerged recently, including genome-wide association study (GWAS) that uses natural plant populations as mapping populations. Further, high-throughput sequencing technology provides access to a large resource of omic data and increases options of host species, GWAS mapping population(s) and effective data analysis strategies for the identification of quantitative genes/loci.

In the past decade, new advances in mechanisms of plant defense to *S. sclerotiorum* infection have been obtained. Further, the recent application of omic methodologies and instrumentation enhances our understanding of defense strategies in important crops. Here, we focused on past studies on mechanisms and strategies employed by plants in coping with this pathogen infection. We also have a particular interest on defense signaling mediated by diverse signaling molecules. Finally, we discussed candidate resistance genes mapped by using High-throughput experiments. This review highlights challenges in defense mechanisms identification, which will broaden our understanding of the genetic architecture underlying this quantitative resistance.

## Plant Defense Mechanisms Against S. *Sclerotiorum*

**Figure 1:**
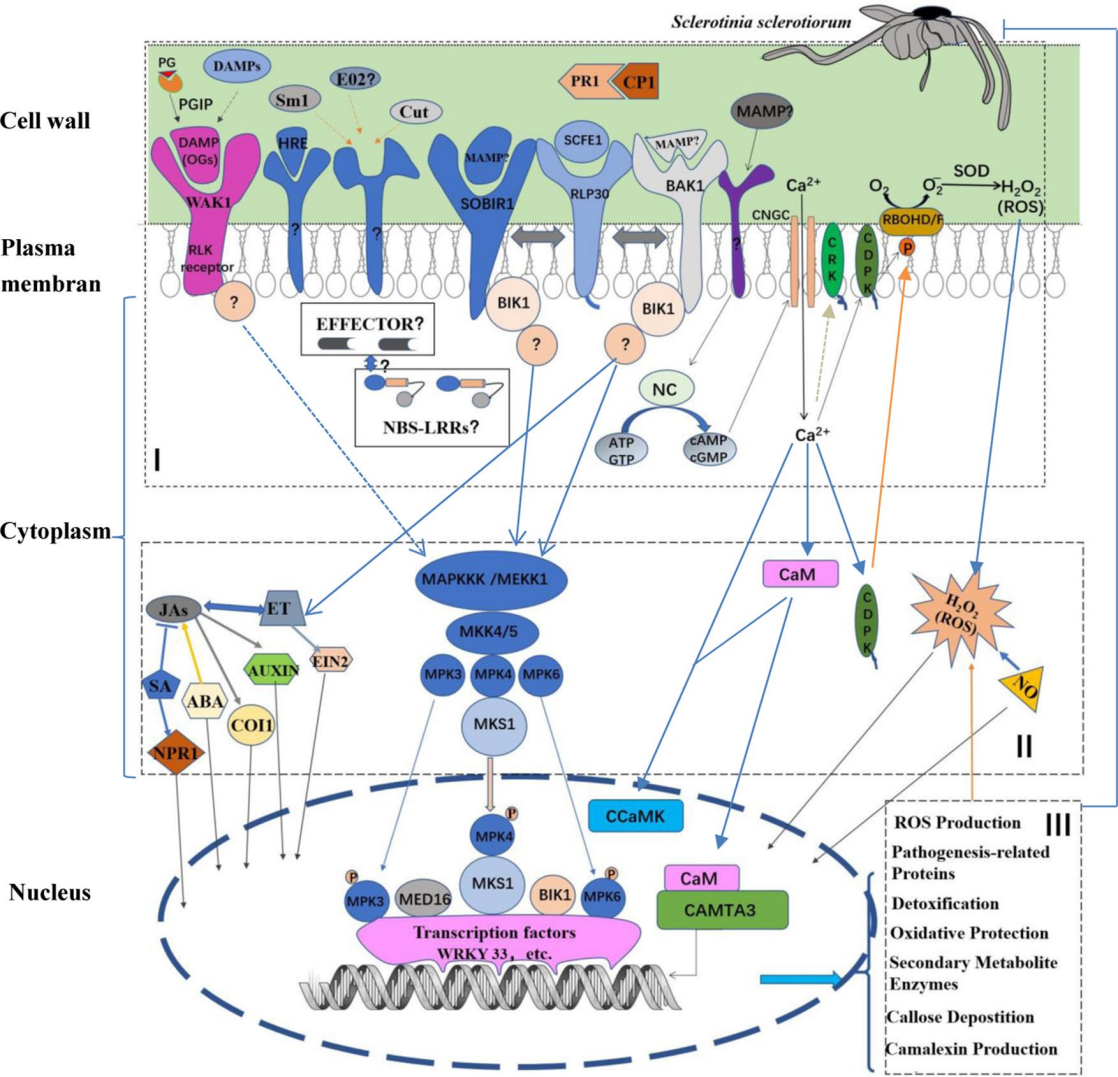
Schematic model of plant innate immune defense process against *Sclerotinia sclerotiorum* infection. In the model, plant defense process can be split into (I) recognition, (II) signal-transduction, and (III) defense response. (I) Plant recognition of MAMPs (or PAMPs) and DAMPs are detected by PRRs in apoplast. The SCFE1–RLP30/SOBIR1/BAK1 recognition, a classical PAMP–PRR recognition mode, is shown. PRRs of other MAMPs and the recognition of OGs, typical DAMPs, by WAK1 remain to be identified. Additionally, potential effectors secreted by *S. sclerotiorum* may recognized by potential NBS-LRRs R protein in cytoplasm. (II) The signal-transduction is performed by the MAP kinase cascade or MAPK-independent pathways. In response to *S. sclerotiorum* infection, the MAPK-independent pathways, such as calcium (Ca) signaling pathway, are also involved. In the Ca signaling pathway, firstly the NC may be activated by unknown PAMP(s) to produce cAMP and cGMP. Subsequently, the two cyclic nucleotides activate Ca^2+^ channels such as CNGCs, resulting in a Ca^2+^ elevation in the cytoplasm. Then the signal of the cytosolic Ca^2+^ elevation is transduced by Ca^2+^ sensor proteins, such as CaMs, CDPKs as well as CRKs. Further, CDPKs phosphorylate and activate RBOHD/F, resulting in ROS accumulation. However, substrates of CRKs are not yet identified under the pathogen infection. Importantly, many signal molecules, including SA, JA, ET, auxin, ABA, NO as well as ROS, play key roles in the signal-transduction. (III) These signals direct various nuclear proteins including transcription factors, transcription activators as well as protein kinases, such as WRKY33, CAMTA3 and CCaMK, to activate specific defense responses, including pathogenesis-related proteins, ROS production, detoxification, oxidative protection, callose deposition camalexin production and other specialized secondary metabolites. Partition of the defense process is generally according to the ref. (Corwin and Kliebenstein, 2017), and the model is expounded in the following text. PR1, pathogenesis-related protein1; MAMPs, microbial associated molecular patterns; DAMPs, damage-associated molecular patterns; PG, polygalacturonases; RLPs, receptor-like proteins; BAK1, BR insensitive1-associated receptor kinase1; SOBIR1, suppressor of BIR1 BIR1-1; WAK1, wall-associated kinase 1; RLK, receptor-like kinase; BIK1, Botrytis-induced kinase1; NBS-LRR, nucleotide-binding site-leucinerich repeat; MAPK, mitogen activated protein kinase; MAPKKKs, MAPK kinase kinases; MAPKKs, MAPK kinases; MKS1, MPK4 substrate 1; SA, salicylate; JAs, jasmonates; ET, ethylene; EIN2, ethylene insensitive 2; ABA, abscisic acid; NO, nitric oxide; NPR1, nonexpressor of *PR1*; COI1, the coronatine- insensitive protein 1, and MED16, mediator complex subunit; NC, nucleotidyl cyclase; CNGC, cyclic nucleotide gated channel; ATP, adenosine triphosphate; GTP, guanosine triphosphate; cAMP, cyclic adenosine monophosphate; cGMP, cyclic guanosine monophosphate; NC, nucleotidyl cyclase; CaM, calmodulin; CDPK, calcium-dependent protein kinase; CRK, CDPK-related kinase; RBOH, respiratory burst oxidase homologue; SOD, superoxide dismutase; CCaMK, Ca and CaM-dependent protein kinase; CAMTA3, CaM-binding transcription activator3.

Facing the attack of pathogens, such as *S. sclerotiorum*, plants need to accurately detect and send timely signal and then appropriately respond to each of the different pathogenic strategies by deploying a range of multifaceted defense response mechanisms (**Figure 1**). Defense responses often begin with either recognition of pathogen (or microbe)-associated molecular patterns (PAMPs or MAMPs) by plant-cell-surface pattern recognition receptors (PRRs) or recognition of the pathogen’s virulence molecules, termed effectors, by a set of plant intracellular resistance (R) gene products, which results in MAMP (or PAMP)-triggered immunity (MTI or PTI) or effector-triggered immunity (ETI), respectively (Jones and Dangl, 2006). In 1971, ETI was also named as gene-for-gene interaction by Flor, (1971). In the plant–*Sclerotinia* pathosystem, ETI (or *R* gene-mediated resistance) has not been observed (Yang et al., 2018). In contrast, the presence of SCFE1 (*Sclerotinia* culture filtrate elicitor1), an elicitor in *S. sclerotiorum* evoking MAMP-triggered immune responses and sensed by RLP30 (Receptor-like protein30), demonstrates the relevance of MTI in resistance to *S. sclerotiorum* (Zhang et al., 2013). RLP30, belonging to a specific class of plasma membrane (PM)-localised receptors, presents all the hallmarks of a cell surface-located RLP with an N-terminal signal peptide, an extracellular domain containing 21 LRRs (which possibly act as the SCFE1 binding site), a single transmembrane domain, and a short cytoplasmic tail of 25 amino acid residues (Zhang et al., 2013). Other elicitors, such as HRE (A heat-released elicitor) (Bertinetti and Ugalde, 1996), SsCut (cutinase) (Zhang et al., 2014) and SsSm1 (a Cerato-platanin family protein) (Pan et al., 2018), have also been identified from *S. sclerotiorum*. Upon infection with *S. sclerotiorum*, these elicitors can inspire the host plant to produce an immune response against this invading pathogen. Interestingly, a fungus-secreted protein, the cerato-platanin protein (CP), acts as a MAMP to elicit plant defense responses (Djonovic et al., 2006; Seidl et al., 2006; Yang et al., 2009; Frias et al., 2011), but *S. sclerotiorum* CP1 (SsCP1) interacts with PR1, overexpression of which shows more resistance to *S. sclerotiorum*, in the apoplast to facilitate infection by *S. sclerotiorum* (Yang et al., 2018). CP also induces synthesis of ROS and triggers local cell death (Pazzagli et al., 1999; Yang et al., 2009; Frias et al., 2011), suggesting that the MTI triggered by CP may be involved in the production of HR, which may also facilitate the infection by *S. sclerotiorum* with necrotrophic lifestyle. In addition, a recent study showed that treatment of *N. benthamiana* with VmE02 (a small cysteine-rich protein), a novel PAMP from the necrotrophic fungus *Valsa mali*, enhances plant resistance to *S. sclerotiorum* and *Phytophthora capsici*. (Nie et al., 2019).

Host damage-associated molecular patterns (DAMPs) also activate protective immune signaling in plants. Oligogalaturonides (OGs) are representative DAMPs. They are released from plant cell wall after infection of pathogens like *Botrytis cinerea* and *S. sclerotiorum*, function as DAMPs, induce plant defense response (Ferrari et al., 2013). OGs are recognized by WAK1 (WALL-ASSOCIATED KINASE 1) who is capable of activating the kinase domain of the elongation factor Tu (EF-TU) receptor (EFR). So OGs seem to be involved in MTI responses to necrotrophic pathogens (Mengiste, 2012). Interestingly, polygalacturonase inhibitor proteins (PGIPs) seem to shift a breakdown process toward generating OGs. In *A. thaliana*, the overexpression of *B. napus PGIP2* inhibited necrotic lesions, but had no long-term effects on *S. sclerotiorum* disease progression (Bashi et al., 2013). Recently, a study showed that the ectopic expression of *OsPGIP2* in rapeseed conferred the resistance to *S. sclerotiorum* at both the seedling and adult stages (Wang et al., 2018).

RLP30 belongs to a specific class of plasma membrane (PM)-localized receptors that carry an extracellular ligand-binding domain, but lack any obvious cytoplasmic signaling-competent moiety (Fritz-Laylin et al., 2005; Wang et al., 2010; Zhang et al., 2013; Liebrand et al., 2014), so following recognition of MAMPs by RLP30, the transduction of the extracellular signals to intracellular targets is dependent on additional signaling partners. Two transmembrane RLKs (receptor-like kinases), BAK1 (Brassinosteroid insensitive1-associated receptor kinase1) and SOBIR1/EVR (SUPPRESSOR OF BIR1-1/EVERSHED) are identified as RLP30 interacting and signaling partners, and are responsible for subsequent signal-transduction to cytoplasmic targets (Zhang et al., 2013). For example, activation of mitogen activated protein kinases (MAPKs), including MAPK 3, 4, and 6, by the SCFE1-containing fraction was impaired in *bak* and *sobir1* mutant plants, indicating that *BAK1* and *SOBIR1* are required for the signal transduction from RLP30 to the intracellular MAPK kinase kinase(s) (MAPKKK). MAPKKK activates the MAP kinase kinase(s) (MAPKK), by which the MAP kinase(s) (MAPK or MPK) is sequential activated by MAPKK. Additionally, SCFE1-dependent production of ethylene (ET), an important signaling molecule, was abolished in *bak1-5* mutant plants, and mutants of *RLP30*, *BAK1*, and *SOBIR1* are more susceptible to *S. sclerotiorum* and the related fungus *Botrytis cinerea* (Zhang et al., 2013). Further, the signaling from RLP30/SOBIR1/BAK1 to MAPKKK might require the RLCK (receptor-like cytoplasmic kinase), because Botrytis-induced kinase1 (BIK1), a RLCK, can cooperate with BAK1 to regulate constitutive immunity and cell death in *A. thaliana* (Eckardt, 2011), which need to be further identified in the pathosystem between *S. sclerotiorum* and its hosts. BIK1 was initially identified as a plasma membrane localized protein (Veronese et al., 2006). A recent study showed that BIK1 can also localize to the nucleus and interact directly with WRKY transcription factors, such as WRKY33, to be involved in the jasmonic acid (JA) and salicylic acid (SA) regulation (Lal et al., 2018).

MAPKs play an important role in signal transduction from cytoplasmic to nucleus, leading to the rapid changes of transcription depending on a series of phosphorylation. It has been reported that the MAP kinase 4 (MPK4) and the WRKY33 transcription factor play an important role in resistance to *S. sclerotiorum* as well as *Botrytis cinerea* (Petersen et al., 2000; Brodersen et al., 2006; Wang et al., 2009; Wang et al., 2014; Wang et al., 2015a). MPK4 is localized in cytoplasm and nuclei, and the MPK4 substrate MKS1 is primarily localized in nuclei (Andreasson et al., 2005). In the absence of pathogens, depending on MKS1, MPK4 can exist in nuclear composited with WRKY33 (Qiu et al., 2008; Wang et al., 2014). Challenge with pathogens or MAMPs leads to the activation of MPK4 and phosphorylation of MKS1. Subsequently, the MKS1–WRKY33 complex is released from MPK4, and WRKY33 activates the expression of *PHYTOALEXIN DEFICIENT 3* (*PAD3*) and *Cytochrome P450 71A13* (*CYP71A13*) (Qiu et al., 2008), both of which are involved in antimicrobial camalexin synthesis (Birkenbihl et al., 2012; Liu et al., 2015). WRKY33 also positively regulates the expression of JA/ET defense pathway marker gene *PLANT DEFENSIN1.2* (*PDF1.2*) and *OCTADECANOID-RESPONSIVE ARABIDOPSIS AP2*/*ERF59* (*ORA59*) (Birkenbihl et al., 2012; Wang et al., 2014; Wang et al., 2015a). However, how WRKY33 is released from the MKS1-WRKY33 complex remains to be determined. The MKS1–WRKY33 complex can exist both before and after phosphatase treatments of MKS1 in *A. thaliana*, and phospho-mimics, non-phosphorylatable, and wild-type forms of MKS1 bind WRKY33 equally well in yeast, suggesting that the complex exists independently of MKS1 phosphorylation (Qiu et al., 2008). It has been shown that WRKY33 can be phosphorylated by MPK3/MPK6 *in vivo* in response to the pathogen infection in *A. thaliana*, which may be a hint for the release of WRKY33. A recent study has shown that *B. napus* MPK3 is a key regulator in defense responses to *S. sclerotiorum* (Wang et al., 2019).

The *A. thaliana* mediator complex subunit MED16 can also physically interact with WRKY33 in yeast and in planta (Wang et al., 2015a). The mediator complex, a multiprotein co-activator scaffold acting as a bridge between RNA polymerase II (RNAPII) and transcription factors, is involved in host transcriptional reprogramming after pathogen challenge, being paramount in the establishment of plant defense (Samanta and Thakur, 2015). Mutations in MED16 subunit abolished induction of JA/ET cross-talk genes and reduced resistance to *S. sclerotiorum* and *B. cinerea* (Kidd et al., 2009; Zhang et al., 2012; Wang et al., 2015a). Thus, these data suggest that WRKY33 seem to be a recruitment center in defense to *S. sclerotiorum*, as well as *B. cinerea*.

In addition to the MAPK pathway, calcium (Ca) signaling pathway has been reported to play important roles in plant defense against *S. sclerotiorum*. A Ca^2+^ elevation in the cytoplasm is known to be an important early event in plant cell perception of pathogen invasion (Dangl et al., 1996; Ma and Berkowitz, 2011). Plant cyclic nucleotide gated ion channels (CNGCs) provide a pathway for Ca^2+^ conductance across the plasma membrane (PM) and facilitate cytosolic Ca^2+^ elevation in response to pathogen signals. In the case of the interaction of plant host with *S. sclerotiorum*, pharmacological assays showed that the putative CNGC activators cGMP (cyclic adenosine monophosphate) and cAMP (cyclic guanosine monophosphate) enhanced resistance of tomato (*Solanum lycopersicum*) against this pathogen (Saand et al., 2015a). Further, two tomato CNGC genes, *SlCNGC1* and *SlCNGC6*, were reported to play a positive role in tomato resistance to *S. sclerotiorum* (Saand et al., 2015b). Interestingly, other CNGC genes such as *SlCNGC17* and *SlCNGC18*, exhibited negative roles in this resistance (Saand et al., 2015a). Transient elevations of the Ca^2+^ concentration in the cytoplasm can be sensed by various Ca^2+^ sensor proteins including calmodulins (CaMs) and calcium-dependent protein kinases (CDPKs) (Ranty et al., 2006; Hamel et al., 2014). It has been reported that a CaM-binding transcription activator CAMTA3 negatively regulated PTI probably by directly targeting *BAK1* under the *S. sclerotiorum* infection (Rahman et al., 2016). In contrast, Ca and CaM-dependent protein kinase (CCaMK), a nuclear-localized protein, positively regulates resistance to *S. sclerotiorum via* promoting H_2_O_2_ accumulation (Wang et al., 2015b). The plasma membrane- and the cytosol-localized CDPKs can also positively regulate H_2_O_2_ accumulation *via* phosphorylating and activating RBOHD/F (Liu and He, 2016). However, knock-down of a set of *CDPKs* did not affect resistance to *S. sclerotiorum* in tomato (Wang et al., 2016). In contrast, *SlCRK6*, a CDPK-related kinase (CRK) that probably localizes at the plasma membrane (Leclercq et al., 2005; Rigo et al., 2013), plays a positive role in this resistance (Wang et al., 2016). The CRK is another type of protein kinase closely related to CDPKs in structure. Unlike CDPKs, however, substrates of CRKs are not yet identified under the pathogen infection. Together, the increasing evidence indicated roles for Ca^2+^ signaling in resistance to *S. sclerotiorum*. Activation of Ca^2+^ channels, such as CNGC, requires the involvement of nucleotidyl cyclase (NC) which generates Ca^2+^ channel activators: cAMP and cGMP, together referred to as the cyclic nucleotide signaling system (Swiezawska et al., 2018). However, how plant NC is activated in response to *S. sclerotiorum* infection remains to be identified.

In fact, a large body of data has implied that plant defense to *S. sclerotiorum* is complex and involves multiple signaling pathways. This is supported by a recent study on the protein profile of *B. napus* in response to *S. sclerotiorum*. The study indicated that plant defense to this pathogen involves various biological processes including redox homeostasis, lipid signaling, calcium signaling, histone, and DNA methylation-mediated transcription regulation and defense-related proteins such as defensin and defensin-like proteins as well as cyanate lyase (Cao et al., 2016a). Moreover, it was reported plant defense to *S. sclerotiorum* is also regulated at miRNA level and probably involves PTGS (Post-Transcriptional Gene Silencing) (Cao et al., 2016b). More recently, glycolate oxidase genes, encoding crucial enzymes in photorespiration, were reported to be involved in resistance to *S. sclerotiorum* (Xu et al., 2018b), suggesting a potential role for photorespiration in this resistance. In addition, defense against *S. sclerotiorum* was found to be stage/phase-associated, and the phytohormones SA, ET, JA and abscisic acid (ABA) likely play an essential, but pathosystem-dependent, role in leaf stage associated resistance (Xu et al., 2018a).

## Signaling Molecules and Their Crosstalk in Plant Response to *S. Sclerotiorum*

Initiation of MTI, as well as ETI, is correlated with a complex network of defense signaling pathways, resulting in defensive cellular responses and changes in expression of thousands of host genes (Zipfel et al., 2006; Zipfel, 2014; Lewis et al., 2015). The hormones, such as JA, SA, ET, ABA, and auxin, function as signaling molecules to transduce the perception signal through a huge and complex signaling network, and then complex defense responses are activated.

Early studies on global profiles of host gene expression in response to infection with *S. sclerotiorum* indicated that genes associated with JA and ET signal are induced, but few of SA responsive genes are identified (Zhao et al., 2007; Zhao et al., 2009). In plant immune responses, SA can antagonize the JA signaling pathway, which has already been identified in plants during *S. sclerotiorum* infection (Wang et al., 2012), and JA and ET are well known to synergistically induce defense responses and cooperate in resistance against pathogens (Thomma et al., 2001; Kunkel and Brooks, 2002; Glazebrook, 2005). However, a study on *Arabidopsis* mutants, including *npr1* (*nonexpressor of PR genes1*) being defective in SA perception, *coi1-2* impaired in JA perception, and *ein2* (*ethylene insensitive*), revealed that defense against *S. sclerotiorum* in *A. thaliana* is dependent on all of SA-, JA- and ET-mediated signaling (Guo and Stotz, 2007). The conclusion is supported by results from *B. napus* (Wang et al., 2012; Nováková et al., 2014), and it is suggested that defense against *S. sclerotiorum* in *B. napus* is associated with a sequential activation of SA and JA signaling (Wang et al., 2012). This can be explained by the new model depicting the lifestyle transition of the pathogen from biotrophic to necrotrophic growth (Kabbage et al., 2015), because it has been suggested that the SA signaling protects against biotrophs, while JA/ET signaling activates defense responses against necrotrophs (Penninckx et al., 1996; Thomma et al., 1998; Glazebrook, 2005), as well as herbivorous insects (Howe and Jander, 2008; War et al., 2012). In favor of a role of ET signaling in this defense, is the recent discovery that *B. napus* MPK3, a positive regulator of ET signaling, positive regulates resistance to *S. sclerotiorum* (Wang et al., 2019).

Roles of SA and JA signaling in defense against *S. sclerotiorum* are challenging. By using three different mutants or transgenic lines impaired in SA production (*sid1/eds5*, *sid2/eds16*, and *nahG*) and one impaired in SA signaling (*npr1-1*), Perchepied et al. (2010) suggested that resistance to *S. sclerotiorum *is not dependent on SA. And they thought that the availability of bioactive jasmonates is not essential for resistance to *S. sclerotiorum*, because *jar1-1*, impaired in biosynthesis of jasmonoyl-L-amino acid, showed a completely wild-type phenotype in response to *S. sclerotiorum* (Perchepied et al., 2010), although *coi1-1* was found to be highly susceptible to *S. sclerotiorum* (Guo and Stotz, 2007). However, studies have shown that SA, BTH (benzothiadiazole, a SA functional analogue) and MeJA application to *B. napus* leaves significantly results in increased resistance to *S. sclerotiorum* (Wang et al., 2012; Nováková et al., 2014). These results suggest that the role of SA or JA signaling in defense against *S. sclerotiorum* may vary depending on the plant species or that possible crosstalk between these signaling pathways and other regulatory pathways is likely to play a role in this defense (Perchepied et al., 2010). For example, an analysis on both jasmonate-dependent and COI1-independent defense responses against *S. sclerotiorum* showed that auxin signaling regulates the COI1-independent defense response pathway and plays an important role in resistance to this pathogen in *A. thaliana* (Stotz et al., 2011).

It has been suggested that ABA signaling would interlink with SA, JA, and ET signaling to affect pathogen resistance *via* a complex interplay of mechanisms (Mauch-Mani and Mauch, 2005; Adie et al., 2007; Kaliff et al., 2007). For example, ABA is required for biosynthesis of JA and expression of JA signaling defense gene in response to infection with *Pythium* spp., a necrotrophic pathogen (Adie et al., 2007). In the case of resistance to *S. sclerotiorum*, *A. thaliana* mutants (*aba3-2*, *abi1-1*, and *abi2-1*), being defective in ABA biosynthesis *or* perception, showed a complete loss of resistance to *S. sclerotiorum* (Guimaraes and Stotz, 2004; Perchepied et al., 2010). ABA-mediated regulation of guard cells is a major mechanism of this defense in these interactions, which is compatible with two views. One is that OA, the essential pathogenicity factor for *S. sclerotiorum*, was concluded to favor infection by both inducing stomatal opening and inhibiting ABA-mediated stomatal closure (Guimaraes and Stotz, 2004; Billon-Grand et al., 2012). Another is that the decreased ambient pH caused by *S. sclerotiorum* infection results in increased synthesis of photoprotective compounds of the xanthophyll cycle that serve as precursors for ABA synthesis, and thus the decrease in *de novo* ABA biosynthesis through decreasing xanthophyll precursors was suggested to account for enhanced plant susceptibility to *S. sclerotiorum* (Zhou et al., 2015). These observations also suggest that ABA signaling may be bi-directionally regulated by both *S. sclerotiorum* and its host plants, which needs to be fully understood by further studies. However, in other two *A. thaliana* mutants (*aba2-3* or *abi5*), they didn’t show any significant changes in their response to *S. sclerotiorum* compared to control plants (Perchepied et al., 2010), suggesting that it remains unclear whether ABA signaling is required for this resistance.

In addition to these hormones, NO (nitric oxide) and ROSs (reactive oxygen species) are important signaling molecules (Kunkel and Brooks, 2002). Nitric Oxide (NO) is rapidly generated after recognition of pathogens (Wendehenne et al., 2004). The NO-impaired mutants showed an extremely susceptible phenotype to *S. sclerotiorum*, revealing the role of NO in resistance to this pathogen (Perchepied et al., 2010). It is suggested that the major role of NO in this resistance is multifaceted, such as regulating defense gene expression, interfering with the ROS signaling pathway, or modulating cell death (Perchepied et al., 2010). It has been shown that inhibition of host cell death by expressing negative regulators of mammalian apoptosis in transgenic tobacco plants leads to markedly enhanced resistance to *S. sclerotiorum* (Dickman et al., 2001); when ROS induction is inhibited, apoptotic-like cell death induced by oxalic acid does not occur and the PCD (programmed cell death) response is required for disease development (Kim et al., 2008). Further, the control of cell death governs the outcome of the *S. sclerotiorum*–plant interaction (Kabbage et al., 2013) and, once infection is established, the necrotrophic *S. sclerotiorum* induces the generation of plant ROS, leading to PCD of host tissue, the result of which is of direct benefit to the pathogen (Williams et al., 2011). Further experiments should be carried out to fully investigate how NO interference with the ROS signaling pathway to inhibit PCD in the context of *S. sclerotiorum*–plant interactions.

## Polygenic Architecture of Quantitative Resistance to *S. sclerotiorum*

Plant immune response to necrotrophs is governed by a complex interplay of minor-effect genes, which results in a full continuum of resistance phenotypes in natural plant populations, designated as quantitative disease resistance (QDR) (Roux et al., 2014). The genetic determinants of QDR are complex, and the underlying genetic components can be common with, but are generally not limited to, PTI and ETI response genes (Iakovidis et al., 2016).

In the case of *S. sclerotiorum*, its host plants, such as the model plant *A. thaliana*, *B. napus* and soybean, show symptoms ranging from high susceptibility to relative tolerance to the pathogen, corresponding to a typical QDR response (Kim et al., 1999; Chen and Wang, 2005; Liu et al., 2005; Perchepied et al., 2010), which involves allelic variation at different quantitative trait loci (QTLs) since the continuous distribution of heritable phenotypes must result from combinations of genetic loci (Corwin and Kliebenstein, 2017). However, although a large body of mapping information on QTLs is available for the QDR to the pathogen (Bert et al., 2002; Zhao and Meng, 2003; Bert et al., 2004; Micic et al., 2004; Micic et al., 2005a; Micic et al., 2005b; Ronicke et al., 2005; Zhao et al., 2006; Yin et al., 2010; Behla, 2017; Wu et al., 2013; Wei et al., 2014), relatively little is known about the molecular basis of QTLs.

Recent research technologies have developed efficient omic tools to better understand the genetic and molecular mechanisms regarding plant QDR to *S. sclerotiorum*. Chalhoub et al. (2014) predicted a total of 181 and 245 putative NBS-LRR resistance genes on the A and C subgenomes of the *B. napus* genome through examining large-scale genomic data. Interestingly, from these genes, Li et al. (2015) found a total of 26 candidate NBS-LRR genes associated with resistance to *S. sclerotiorum* through integrating and comparing QTLs for resistance to this pathogen from previous mapping efforts. Correspondingly, a bioinformatic study revealed that the *S. sclerotiorum* genome encodes a large set of candidate effector proteins (Guyon et al., 2014). *R* gene-mediated resistance commonly results in rapid cellular desiccation and death at the site of attempted infection that constitutes a hypersensitive response (HR) (Wright and Beattie, 2004; Dodds and Rathjen, 2010). Consistently, some researchers claimed that they observed HR-like lesions on *S. sclerotiorum*-inoculated stems (Uloth et al., 2013; Uloth et al., 2015; Ming et al., 2016) or cotyledons (Garg et al., 2008; Garg et al., 2010; Uloth et al., 2014; Ge et al., 2015). These data suggested that *R*-mediated resistance seems to exist in the interaction of *B. napus* with *S. sclerotiorum*. Considering the continuous distribution of disease resistance phenotype in host populations, a typical characterization of QDR, roles of these candidate *R* genes in this resistance need to be confirmed further, because *R*-mediated resistance can be seen as an extreme of the phenotypic spectrum, in which the switch from susceptibility to resistance in plant populations is reduced to a minimum of detectable transition states (Roux et al., 2014).

With the development of high-throughput sequencing technology, genome-wide association study (GWAS) based on linkage disequilibrium (LD), has emerged as an important tool for identifying small-to-moderate effect loci associated with resistance to *S. sclerotiorum*. For example, based on GWAS, two recent studies identified two QDR genes, coding for the POQR prolyl oligo peptidase and the actin-related protein complex isoform 4, respectively, for *S. sclerotiorum* in the model plant *A. thaliana* (Badet et al., 2017; Badet et al., 2019). However, in most cases of crops, GWAS cannot lead directly to the gene(s) at a given locus because of insufficient marker density and linkage disequilibrium. Thus, GWAS data are usually combined with other omic experiments, such as microarray study, RNA sequencing (RNAseq), to interpret the results, which can increase the confidence in identifying candidate defense-associated (CDA) genes (Corwin and Kliebenstein, 2017). To date, this approach has been employed to interpret GWAS results associated with resistance to *S. sclerotiorum* in two crops, *B. napus* and soyabean. On the basis of data from these reports from 2015–2018 in the two crops, we focus on these candidate genes related with defense mechanisms, to our knowledge (**Table 1**), and find out the following features in resistance to *S. sclerotiorum*. Additionally, bean CDA genes identified by using a QTL meta-analysis also are considered (**Table 1**).

- Resistance to *S. sclerotiorum* is determined by minor QTLs. These phenotypic contributions of the GWAS-identified loci was low, with each locus explaining less than 10% of the observed phenotypic variance in resistance to *S. sclerotiorum*. This is supported by QTL mapping in which resistance to *S. sclerotiorum* is a trait with very complex genetic underpinnings determined by multiple minor QTLs.
- The predominant group of genes linked to GWAS-identified loci as potential causal genes were those involved in downstream defense responses including pathogenesis-related proteins, ROS production, detoxification, oxidative protection and secondary metabolite enzymes. This implies that QDR to *S. sclerotiorum* is a function of a variety of cellular processes and not simply pathogen-detection and signal-transduction.
- Interestingly, many potential resistance (*R*) genes are identified as potential causal genes by GWAS. It has been suggested that upstream signaling components, such as R protein, in the plant pathogen response are typically encoded by medium-to-large-effect loci, which is supported by the large number of studies that investigated the quantitative genetics of wheat resistance to wheat stripe rust (Fu et al., 2009), *Arabidopsis* resistance to *Xanthomonas campestris* (Huard-Chauveau et al., 2013) and *Fusarium oxysporum* (Diener and Ausubel, 2005; Shen and Diener, 2013) and rice resistance to *Magnaporthe oryzae* (Ballini et al., 2008; Miah et al., 2013; Kang et al., 2016; Raboin et al., 2016). In the case of resistance to *S. sclerotiorum*, all of these potential candidate R genes are located in small-effect loci. Is ETI response to *S. sclerotiorum*, if it exists, a quantitative trait? A recent report has shown that an ETI response in *Arabidopsis* resistance to *Pseudomonas syringae*, a hemi-biotroph, is a quantitative trait, in which a single effector, HopAM1, was used to identify quantitative natural variation in the response to this effector (Iakovidis et al., 2016). Considering the identified large set of candidate effector protein from *S. sclerotiorum *genome (Guyon et al., 2014) and a potential double feeding lifestyle of *S. sclerotiorum*, it is possible to use these candidate effectors to test if there is quantitative variation in ETI response to *S. sclerotiorum* signals.
- In the case of *B. napus*, an amphidiploid formed by interspecific hybridization of the two diploid species *B. rapa* (AA,n = 10) and *B. oleracea* (CC, n = 9), the GWAS data showed that the majority of potential causal genes for the pathogen were identified in the C genome (C9 and C6), but not in A genome (**Table 1**), although it has been known that putative resistance-related genes in C genome also are observed in the syntenic region on A genome (Mei et al., 2013). These observations suggested that *B. oleracea*, not *B. rapa*, may be a good source of QDR genes for *S. sclerotiorum*. Further, It has been reported that a few Chinese *B. oleracea* var. capitata genotypes exhibit high level stem and leaf resistances to *S. sclerotiorum* (Mei et al., 2011; Ming et al., 2016). Contrastly, there is no genome specificity of QDR genes for this pathogen in soybean.
- Comparing with existing biparental populations, the GWAS populations use more lines and also utilize the increased number of meiotic generations to provide increased recombination and potentially increased mapping resolution (Nordborg et al., 2002; Nordborg et al., 2005; Nordborg and Weigel, 2008; Atwell et al., 2010; Alonso-Blanco et al., 2016). However, so far, the GWAS-identified loci/genes for resistance to *S sclerotiorum* in each analysis collectively explained a small portion of the phenotypic variation, and few loci/genes could be detected repeatedly in different populations for the same species (**Table 1**). This suggests that these GWASs is still largely underpowered given the number of accessions and the effect of residual population structure or adaptive genetic variation unaccounted for in these studies (Platt et al., 2010; Brachi et al., 2015). Thus, future studies with even more powerful populations are required.

**Table 1:** Candidate defense-related genes mapped by genome-wide association study (GWAS) combined with other omic experiments.

Tag	Group	Related Role	Plant	Gene	Protein	Annotation	References
I	Recognition	Recognition of MAMPs	*Brassica napus*	*BnaC06g24200D*	A Leucine-rich receptor-like protein kinase family protein	A RLP-like kinase	(Wu et al., 2016)
I	Recognition	Recognition of MAMPs	*Glycine max*	*Glyma13 g03360*	A PR5-like receptor kinase	A serine/threonine receptor kinase	(Zhao et al., 2015)
I	Recognition	Recognition of MAMPs	*Phaseolus vulgaris*	*Phvul.008G173600*	A Receptor-like protein	A RLP	(Vasconcellos et al., 2017)
I	Recognition	Recognition of DAMPs	*Brassica napus*	*BnaC08g16900D*	A Wall-associated kinase family protein	The cell wall associated protein	(Wu et al., 2016)
I	Recognition	Recognition of DAMPs	*Brassica napus*	*BnaC06g24700D*	The Polygalacturo- nase2 (PG2)	Cell wall modification	(Wu et al., 2016)
I	Recognition	Recognition of DAMPs	*Glycine max*	*Glyma.18G116400*	A probable PG	Cell wall modification	(Wei et al., 2017)
I	Recognition	Recognition of DAMPs	*Glycine max*	*Glyma.18G117100*	A cellulose synthase	Cell wall modification	(Wei et al., 2017)
I	Recognition	Recognition of DAMPs	*Glycine max*	*Glyma.05G044000*	A pectate lyase	Cell wall modification	(Wen et al., 2018)
I	Recognition	Recognition of DAMPs	*Phaseolus vulgaris*	*Phvul.001G236600*	A wall-associated receptor kinase protein	Recognizing cell wall changes	(Vasconcellos et al., 2017)
I	Recognition	Recognition of the pathogen effectors	*Brassica napus*	*BnaC06g30610D*	A teucine-rich repeat (LRR) family protein	The R protein	(Wei et al., 2016)
I	Recognition	Recognition of the pathogen effectors	*Brassica napus*	*BnaC06g24000D*	A TIR-NBS class protein	The R protein	(Wu et al., 2016)
I	Recognition	Recognition of the pathogen effectors	*Brassica napus*	*BnaC06g24010D*	A TIR-NBS-LRR class	The R protein	(Wu et al., 2016)
I	Recognition	Recognition of the pathogen effectors	*Glycine max*	*Glyma.09G062100*	A LRR family protein	The R protein	(Wen et al., 2018)
I	Recognition	Recognition of the pathogen effectors	*Glycine max*	*Glyma.09G062100*	NB-ARC domain protein	Regulating R protein	(Wen et al., 2018)
I	Recognition	Recognition of the pathogen effectors	*Glycine max*	*Glyma.16G135200*	A NB-ARC domain protein	Regulating R protein	(Wen et al., 2018)
I	Recognition	Recognition of the pathogen effectors	*Glycine max*	*Glyma.16G135500*	A NB-ARC domain protein	Regulating R protein	(Wen et al., 2018)
I	Recognition	Recognition of the pathogen effectors	*Glycine max*	*Glyma.16G159200*	A NB-ARC domain protein	Regulating R protein	(Wen et al., 2018)
I or II	Recognition or Signal transduction	?	*Brassica napus*	*BnaC08g16970D*	A Protein kinase superfamily protein	?	(Wu et al., 2016)
I or II?	Recognition or Signal transduction	?	*Glycine max*	*Glyma.14G049600*	A phosphatase	?	(Wen et al., 2018)
II	Signal transduction	Receives the signals from PRRs	*Brassica napus*	*BnaC04g40820D*	The MAPKKK14	The MAPK cascade	(Wu et al., 2016)
II	Signal transduction	Signaling	*Brassica napus*	*BnaC04g40340D*	A NAD(P)-binding Rossmann-fold super family protein	Systemic acquired resistance (SAR)	(Wu et al., 2016)
II	signal transduction	SA signaling	*Glycine max*	*Glyma.01G104100*	The isochorismate synthase	Synthesis of salicylic acid	(Wei et al., 2017)
II	Signal transduction	JA signaling	*Glycine max*	*Glyma.16 g134400*	The carboxyl methyltransferase	JA signaling	(Wen et al., 2018)
II	Signal transduction	JA signaling	*Phaseolus vulgaris*	*Phvul.001G240400*	The coronatine- insensitive protein 1 (COI1)	The jasmonate receptor	(Vasconcellos et al., 2017)
II	Signal transduction	ET signaling	*Brassica napus*	*BnaC06g24360D*	A Ethylene-responsive transcription factor (ERF73)	ET signaling	(Wu et al., 2016)
II	Signal transduction	ET signaling	*Phaseolus vulgaris*	*Phvul.002G055700*	A ERF	ET signaling	(Vasconcellos et al., 2017)
II	Signal transduction	ET signaling	*Phaseolus vulgaris*	*Phvul.002G055800*	A ERF	ET signaling	(Vasconcellos et al., 2017)
II	Signal transduction	ET signaling	*Phaseolus vulgaris*	*Phvul.006G183100*	A ERF	ET signaling	(Vasconcellos et al., 2017)
II	Signal transduction	ET signaling	*Phaseolus vulgaris*	*Phvul.006G183200*	A ERF	ET signaling	(Vasconcellos et al., 2017)
III	Defense response	The PR proteins	*Brassica napus*	*BnaC06g30470D*	The β-1,3-glucanase	The PR-2 family protein	(Wei et al., 2016)
III	Defense response	The PR proteins	*Brassica napus*	*BnaC04g40020D*	A PR thaumatin super family protein	The PR protein	(Wu et al., 2016)
III	Defense response	secondary metabolite	*Glycine max*	*Glyma13 g04031*	The MYB domain protein 33	Controlling secondary metabolism	(Zhao et al., 2015)
III	Defense response	secondary metabolite	*Glycine max*	*Glyma.18G113400*	A putative MYB transcription factor	Controlling secondary metabolism	(Wei et al., 2017)
III	Defense response	Secondary metabolite	*Phaseolus vulgaris*	*Phvul.005G115500*	A MYB domain protein	Controlling secondary metabolism	(Vasconcellos et al., 2017)
III	Defense response	Secondary metabolite enzyme	*Brassica napus*	*BnaA08g19770D*	A glucosidase	The cleavage of the glucosid	(Wei et al., 2016)
III	Defense response	The secondary metabolite enzyme	*Brassica napus*	*BnaC04g41120D*	A cinnamate-4- hydroxylase (C4H)	The biosynthesis of monolignols and anthocyanins	(Wu et al., 2016)
III	Defense response	The secondary metabolite enzyme	*Brassica napus*	*BnaC04g41130D*	A C4H	The biosynthesis of monolignols and anthocyanins	(Wu et al., 2016)
III	Defense response	The secondary metabolite enzyme	*Glycine max*	*Glyma.04G198000*	A acyltransferase	Secondary metabolism biosynthesis	(Wen et al., 2018)
III	Defense response	The secondary metabolite enzyme	*Glycine max*	*Glyma.16G158100*	A UDP- glucosyltransferase	Secondary metabolism biosynthesis	(Wen et al., 2018)
III	Defense response	The secondary metabolite enzyme	*Brassica napus*	*BnaC06g37610D (BnaC.IGMT5.a)*	An indole glucosinolate methyltransferase	Secondary metabolite	(Wu et al., 2013; Wei et al., 2016)
III	Defense response	detoxification	*Glycine max*	*Glyma.16G158100*	A glucuronosyl- transferases	Detoxification mechanism	(Wen et al., 2018)
III	Defense response	detoxification	*Glycine max*	*Glyma.06G106100*	An oxalate exchanger- related (OER) protein	Detoxification of oxalic acid	(Wen et al., 2018)
III	Defense response	detoxification	*Glycine max*	*Glyma.07G218800*	A protein encoded by an OER gene that do not overlap with GWAS-identified loci	Detoxification of oxalic acid	(Wen et al., 2018)
III	Defense response	detoxification	*Glycine max*	*Glyma.13G087200*	A protein encoded by an OER gene that do not overlap with GWAS-identified loci	Detoxification of oxalic acid	(Wen et al., 2018)
III	Defense response	detoxification	*Glycine max*	*Glyma.19G159000*	A protein encoded by an OER gene that do not overlap with GWAS-identified loci	Detoxification of oxalic acid	(Wen et al., 2018)
III	Defense response	detoxification and oxidative protection	*Glycine max*	*Glyma.01G106000*	A tau class glutathione S-transferase (GST) protein	xenobiotic detoxification, reduction or oxidative protection	(Wei et al., 2017)
III	Defense response	Oxidative protection	*Brassica napus*	*BnaC04g40550D*	The GST tau4 (GSTU4)	An antioxidant defense	(Wu et al., 2016)
III	Defense response	Oxidative protection	*Brassica napus*	*BnaC04g40560D*	The GSTU3	An antioxidant defense	(Wu et al., 2016)
III	Defense response	Antioxidant	*Brassica napus*	*BnaC06g31020D*	A GSTU protein	An antioxidant defense	(Wei et al., 2016)
III	Defense response	Antioxidant	*Brassica napus*	*BnaC06g31030D*	A GSTU protein	An antioxidant defense	(Wei et al., 2016)
III	Defense response	Antioxidant	*Brassica napus*	*BnaC06g31040D*	A GSTU protein	An antioxidant defense	(Wei et al., 2016)
III	Defense response	ROS production	*Phaseolus vulgaris*	*Phvul.003G164600*	The peroxidase	ROS accumulation	(Vasconcellos et al., 2017)
III	Defense response	Controlling HR	*Glycine max*	*Glyma.06G107800*	The serine hydroxyl methyltransferase	Controlling HR	(Wen et al., 2018)
III	Defense response	Cell cycle, cell autophagy	*Glycine max*	*Glyma13 g04020*	A member of the RINT-1/TIP-1 family	Radiation-induced checkpoint control, Golgi transport	(Zhao et al., 2015)
III	Defense response	Unknown	*Brassica napus*	*BnaC04g40700D*	The Zinc finger (C2H2 type) family protein	The transcription factor	(Wu et al., 2016)
III	Defense response	Unknown	*Brassica napus*	*BnaC06g30580D*	The DHHC-type zinc finger protein (ZFP)	The transcription factor	(Wei et al., 2016)
Unknown	Unknown	Defense- associated proteins	*Brassica napus*	*BnaC06g30160D*	The ß-xylosidase	Hydrolysis reaction of xylogucan ologosaccharides	(Wei et al., 2016)
Unknown	Unknown	Defense- associated proteins	*Glycine max*	*Glyma.11G084200*	A GRIP-like protein	Targeting the golgi	(Wei et al., 2017)
Unknown	Unknown	Defense- associated proteins	*Glycine max*	*Glyma.09 g281900*	A O-methyltrans- ferase	O-methyltrans- ferase	(Wen et al., 2018)

## Conclusions

As *S. sclerotiorum* has the broad host range, diverse infection modes and potential double lifestyle of nutrient acquisition, scientists have been trying to dissect the *S. sclerotiorum*–host interaction and to understand the mechanisms of QDR to the pathogen. Current knowledge on plant defense to *S. sclerotiorum* has set up basic framework including recognition of the pathogen, signaling and defense response, but a lot of effort will be needed to enrich the contents. Pyramiding the QDR genes appears to be a promising strategy for durable resistance, but considering the pyramiding power in the gene number, key question is which mechanisms play an important role in this QDR. Thus, the assessment of the phenotypic contribution of QDR genes requires robust and accurate methods. The choice of a truly quantitative readout, which can be precisely and reliably measured, may be essential for both the accuracy of QTL mapping and the validation of QDR gene function. High-throughput omic technologies will broaden the choice of more powerful mapping population(s) and data analysis strategies available for accurate and reliable QDR gene identification.

## Author Contributions

ZW conceptualized and drafted the manuscript, with additions from X-LT, L-YM, JC, Y-LL, L-ND, K-MZ, and Y-HY. The figure was made by L-YM and ZW, and the table was prepared by ZW. All authors read and approved the final manuscript.

## Funding

The authors acknowledge funding support from National Natural Science Foundation of China (Grant Nos. 31771836).

## Conflict of Interest

The authors declare that the research was conducted in the absence of any commercial or financial relationships that could be construed as a potential conflict of interest.
